# Utilizing Analytics to Identify Trends in Residency Program Website Visits

**DOI:** 10.7759/cureus.6910

**Published:** 2020-02-07

**Authors:** Sean Dyer, Bradley Dickson, Neeraj Chhabra

**Affiliations:** 1 Emergency Medicine, Cook County Health, Chicago, USA

**Keywords:** website, residency, application, medical student, emergency medicine

## Abstract

Introduction

Most medical residency training programs maintain websites to provide content for audiences including current residents, prospective residents, and medical students. This study seeks to characterize when and how a residency program website is being accessed in order to identify the primary audiences to provide appropriate and timely content.

Methods

The authors examined website analytics at a large urban Accreditation Council for Graduate Medical Education (ACGME)-accredited post-graduate year (PGY)1-PGY4 emergency medicine residency training program website. Analytics were performed from July 1, 2016 to June 30, 2018 with daily traffic cataloged along with referral sources, page views, and device type used to access the website. The top five dates by daily traffic were further analyzed with daily traffic trends during the residency interview season.

Results

There was an average of 45.8 unique visitors daily with 261.5 daily page views. Computer (67.2%), mobile device (29.6%), and tablet (3.1%) were the most common devices used for viewing. The most popular content areas by page-view were “people” (68,987 visits), “home” (38,569), “clinical curriculum” (35,556), and “medical students” (14,461). The five most-visited dates were all related to application processes including the opening of the Visiting Student Application Service (VSAS), the Electronic Residency Application Service (ERAS), and Match Day. During the interview season, peak visits occurred the dates immediately preceding interview dates.

Conclusion

Residency program websites appear to be accessed most commonly by medical students and prospective residency applicants. Website managers should take the needs of these audiences into account and provide appropriate content to maximally inform prospective residency program candidates.

## Introduction

To keep pace with the rapid evolution in web-based communication and marketing over the past three decades, most medical residency training programs have created and maintained program-specific websites. There is considerable variability in the presence and quality of information that is available on these websites, especially as they contend with the need to provide content for multiple different audiences such as current residents, prospective residents, and medical students [1]. In industries outside of medical training, the task of maintaining websites to promote goods and services is a job unto itself that is often subcontracted to third parties and involves entire teams dedicated to uploading digital content. Significantly fewer resources are available to medical training programs for creating and maintaining websites. Often, those tasked with managing these websites must balance this responsibility with numerous other clinical and administrative tasks. As a result, many residency program websites fail to offer comprehensive information related to their residency program or fail to have a website at all [2-6].

Residency program-specific website managers must understand how, when, and why their website is being accessed to allow for efficient web-based communications. Unfortunately, little is known regarding when and for what purposes residency program-specific websites are being accessed. Such data can be utilized to create an efficient model for editing a program’s website and prioritize the update of commonly accessed content in a timely manner. Thus, the objective of our study was to describe trends in the access patterns of a residency program-specific website.

## Materials and methods

This study was a retrospective analysis using website analytics at one large urban Accreditation Council for Graduate Medical Education (ACGME)-accredited post-graduate year (PGY1)-PGY4 emergency medicine residency training program with 68 residents in training and rotations for both third and fourth-year medical students. Website analytics of the program website were performed using Squarespace™ across two academic years: from July 1, 2016 to June 30, 2018 [7]. Daily traffic was cataloged along with referral sources, page views, and device type used to access the website. The top five dates by daily traffic were specifically analyzed along with daily traffic trends during the residency interview season (October to February).

## Results

Over the 24-month study period, there was an average of 45.8 unique visitors daily with 261.5 page views per day. Computer access accounted for 67.2% of website visits, mobile device for 29.6% of visits, and tablet for 3.1%. Google searches accounted for 49.3% of visits, direct uniform resource locator entry for 41.8%, and Bing searches for 2.4%. The remaining 6.5% of visits were via other sources. The majority of visits (94.2%) originated from within the United States with less than 1% from each of Canada, India, the Republic of Korea, the United Kingdom, Russia, and other countries.

**Figure 1:**
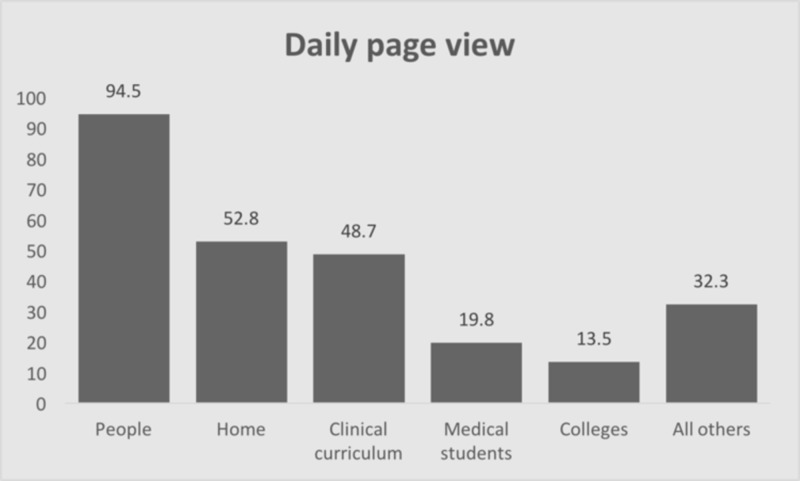
Daily website page views by page

**Figure 2:**
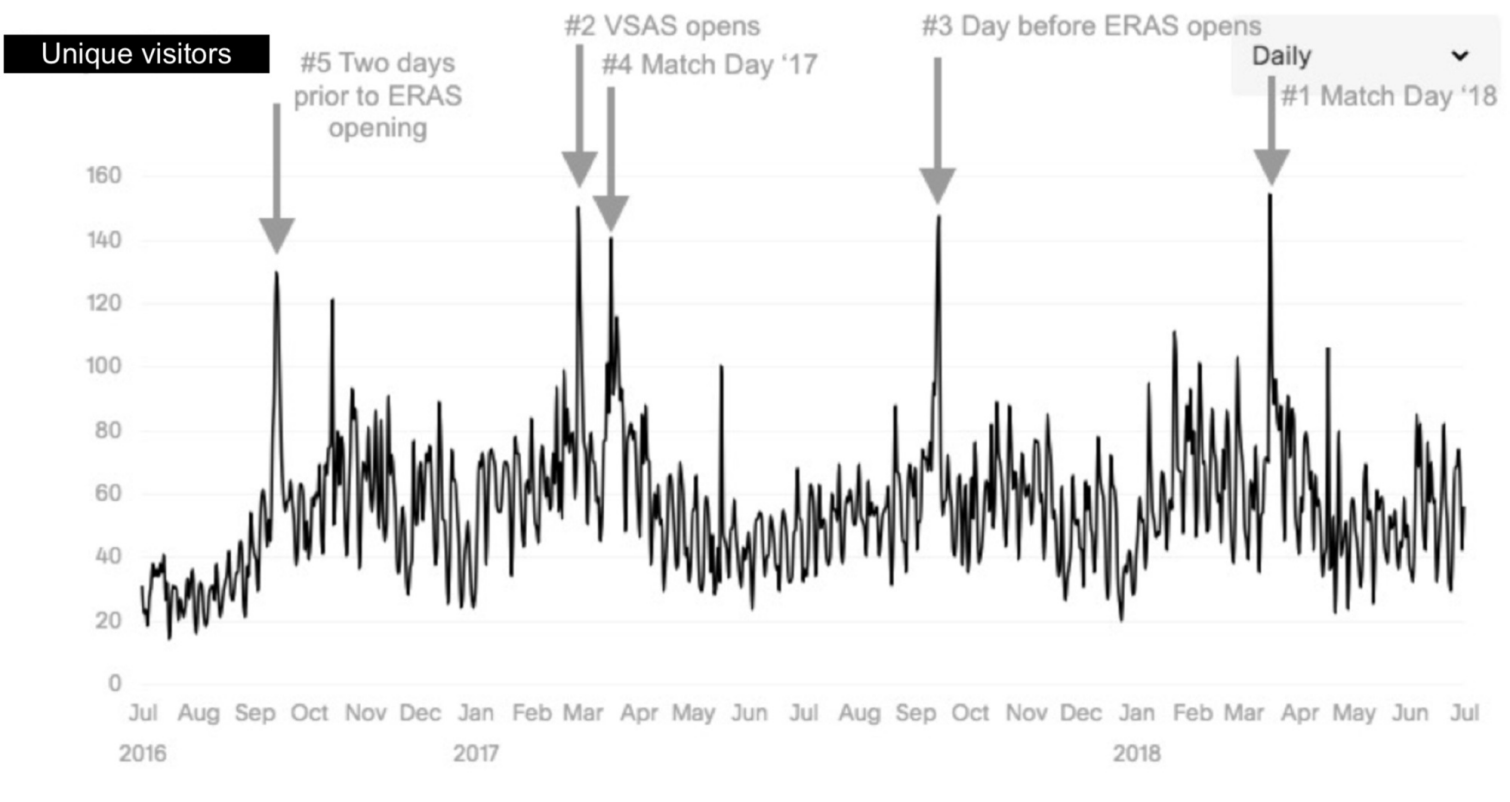
Most-visited dates by daily website traffic ERAS: Electronic Residency Application Service, VSAS: Visiting Student Application Service

**Figure 3:**
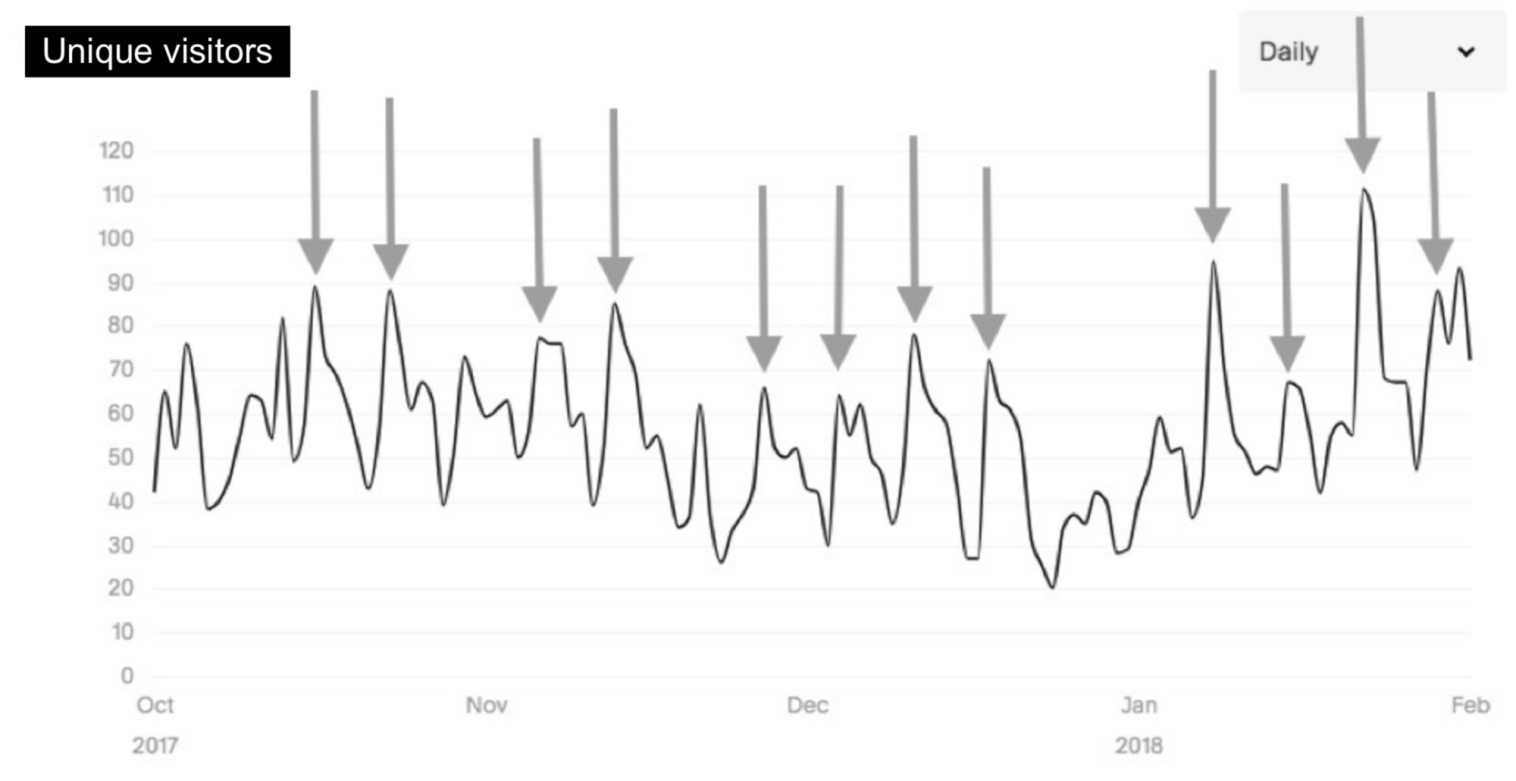
Daily viewing trends during residency interview season: October to February Interview dates indicated by arrows

The most popular content by page-view (Figure 1) were “people” (68,987 visits), “home” (38,569), “clinical curriculum” (35,556), and “medical students” (14,461). The five most-visited dates (Figure 2) were all related to medical student application processes including the opening of the Visiting Student Application Service (VSAS), the Electronic Residency Application Service (ERAS), and Match Day. During the interview season, peak visits occurred on the days immediately preceding a residency program interview date (Figure 3).

## Discussion

Peak access to the residency program-specific website was related to medical student and prospective resident application processes along with Match Day. The days immediately preceding interview dates also corresponded to a sharp increase in website traffic. Medical students and potential residency program applicants are likely significant consumers of residency program-specific website content given peak website traffic tended to parallel medical student’s fourth year away rotation and residency application dates. Website managers should consider this primary audience when creating content for their websites and make the content accessible and pertinent to medical students and prospective residency applicants. 

While computers accounted for the majority of website traffic, about one-third of visits were via a mobile device or tablet. Website managers should thus ensure residency program websites are as mobile device compatible as possible. Additionally, most website traffic was directed via a search engine rather than a direct link from databases such as the Fellowship Residency Electronic Interactive Data Access System (FREIDA) or the ACGME. This finding was not unexpected as these databases have variation in link access to residency program-specific websites [8]. Programs should ensure their websites are easily discoverable through simple keywords used in search engines to increase the likelihood that potential applicants are directed to the correct website.

Data on peak traffic dates can help influence decisions about when website edits should take place. According to our study, this is ideally prior to the opening of the application cycles of VSAS and ERAS. As residency program website managers typically work with limited resources and often balance website-related responsibilities with other administrative or clinical duties, it is critical that changes to the website occur efficiently and in a way that will maximize the impact on website viewership.

With regard to page-view data, the most popular content accessed was the “people” section of the website. This section includes photographs of current residents and faculty and information regarding their medical school and other training. Efforts to keep these sections up to date and include information pertinent to prospective applicants should be prioritized. Interestingly, information regarding the program’s curriculum was accessed much less often, at about half the frequency as the “people” section, which underscores the value prospective applicants may place on the backgrounds of their potential colleagues.

## Conclusions

Residency program-specific websites appear to be accessed most commonly by medical students and prospective residency program applicants. Peak access times tended to cluster around medical student dates of importance such as the VSAS and ERAS application opening dates, Match Day, and the days prior to residency program interview dates. Residency program website managers can use these data to better understand the primary audiences of their residency program websites and determine when to update and upload new content to best inform and attract prospective residency program candidates.
